# Education Research: Online EEG Learning Platforms for Resident and Fellow Education

**DOI:** 10.1212/NE9.0000000000200356

**Published:** 2026-09-01

**Authors:** Quoc Bao Nguyen, Gina Kayal, Ram Mani, Jay R. Gavvala, Zulfi Haneef, Sarah Durica

**Affiliations:** 1 Department of Neurology, University of Oklahoma Health Campus, Oklahoma City;; 2 Department of Neurology, Baylor College of Medicine, Houston, TX;; 3 Department of Neurology, Rutgers Robert Wood Johnson Medical School, New Brunswick, NJ;; 4 Department of Neurology, UTHealth Houston McGovern Medical School, Houston, TX;; 5 Neurology Care Line, Michael E. DeBakey VA Medical Center, Houston, TX; and; 6 Neurorehabilitation Service Line, Oklahoma City VA Medical Center, Oklahoma City.

## Abstract

**Background and Objectives:**

EEG interpretation is a core neurology competency, but instruction and supervised review vary across programs. Online platforms may supplement training, but their design and functionality are not well characterized. We reviewed publicly accessible online EEG platforms for residents and fellows.

**Methods:**

We conducted a scoping review of publicly available online EEG learning platforms using a multipronged identification strategy (June 2025 to January 2026) that included independent web searches by multiple reviewers, targeted screening of professional society and organizational websites, review of EEG education literature and conference materials, author knowledge, and AI-assisted searching using ChatGPT (OpenAI) to generate candidate platform names and search terms. Platforms were eligible if they were stable, web-based educational environments primarily intended to teach EEG interpretation or EEG-related clinical neurophysiology, with structured organization and at least 1 active learning feature beyond one-way media delivery. Two reviewers independently evaluated platform features using a standardized charting form covering educational delivery (accessibility, interactivity, assessment, feedback, and program integration) and EEG technical functionality (content breadth, pediatric/neonatal availability, scrolling and manipulation, and intensive care unit [ICU]/intraoperative monitoring [IOM]/quantitative EEG [qEEG] coverage). When contact information was available, creators of free-to-access platforms were contacted to verify platform features.

**Results:**

Of 16 sources identified, 1 standalone video-sharing channel was excluded, leaving 15 platforms. Ten (67%) were free. Feedback was available on 12 (80%): automated on 9 (60%) and personalized on 3 (20%); all personalized feedback required payment. Only 1 platform (7%) reported trainee progress to a program director. Among free platforms, all included adult EEG examples; 7 (70%) included pediatric content, 4 (40%) neonatal content, 4 (40%) EEG manipulation, 6 (60%) ICU EEG, 3 (30%) qEEG, and 1 (10%) IOM.

**Discussion:**

Online EEG learning platforms provide accessible, scalable foundational instruction but vary in functionality. Gaps include limited pediatric and neonatal content, restricted EEG manipulation, and limited personalized feedback or program-level integration. For training programs, these findings support using online platforms to standardize foundational exposure while integrating them into hybrid curricula that combine self-directed learning with supervised case review, expert feedback, and milestone-based assessment. Future platforms, including artificial intelligence-enabled tools, may further personalize EEG education by tracking learner progress and recommending targeted cases or modules.

## Introduction

EEG interpretation is a core skill in neurology training, yet previous work suggests that residency EEG education remains inconsistent and often insufficiently assessed.^1^ Surveys of US adult neurology residencies and US/Canadian child neurology and neurodevelopmental disabilities programs found substantial variation in required EEG rotations, EEG volume, and teaching structure, with most programs not using objective measures to assess EEG competence or milestones; insufficient EEG exposure was a leading reported barrier.^1,2^

More recent competency-based work has tried to address these shortcomings, but important gaps remain. Existing milestones do not specify which EEG findings trainees should master, prompting development of a “must-know” list of routine EEG findings for adult and child neurology residents.^3^ An online competency-based routine EEG examination subsequently showed that resident accuracy improves with increasing EEG exposure. Although more than 8 weeks of training may be needed to approach expert-level performance, confidence seems to improve more gradually than accuracy.^4^ Although these data are primarily North American, EEG education is needed globally and online EEG platforms can reach trainees across programs and countries with variable access to EEG volume, faculty expertise, and structured curricula.^5^

Across visually intensive diagnostic specialties such as radiology, pathology, and electrocardiography, online educational platforms have increasingly been used to supplement clinical training by providing broad access to image-based learning, case exposure, self-assessment, and asynchronous instruction. EEG interpretation similarly relies on visual pattern recognition and repeated exposure to diverse findings.^6-8^ In this context, online EEG learning platforms may help fill persistent educational gaps by expanding access to structured content, self-paced practice, and scalable teaching resources across programs in the United States and internationally. Previous literature suggests that e-learning and virtual teaching can improve EEG test performance, particularly in settings with limited faculty expertise, teaching time, or EEG exposure.^9,10^ At the same time, online tools are best viewed as supplements rather than replacements for hands-on supervised interpretation and standardized assessment.^9,11^ Deliberate practice requires feedback, correction, and refinement; without expert review, unsupervised practice may reinforce inaccurate interpretation habits.^12^ Online platforms can increase exposure and practice, but skill acquisition requires more than repetition.

Publicly accessible online EEG learning platforms relevant to neurology resident and fellow education have not been systematically characterized. Our study therefore sought to identify these platforms and characterize their educational design, interactivity, assessment and feedback features, and EEG-specific technical functionality relevant to trainee learning.

## Methods

### Study Design

We conducted a scoping review to identify, map, and characterize online EEG learning platforms relevant to neurology resident and fellow education. A scoping approach was chosen to describe the breadth of available resources and their educational and technical features rather than to evaluate educational effectiveness or learner outcomes. We reported methods in accordance with the Preferred Reporting Items for Systematic Reviews and Meta-analyses extension for Scoping Reviews.

### Protocol and Registration

Methods were defined a priori by the study team. A formal protocol was not prospectively registered.

### Review Questions

We sought to answer the following questions: (1) what publicly accessible online platforms exist to support EEG interpretation education for neurology trainees? (2) How do platforms differ in educational delivery (access, interactivity, assessment, feedback, and program integration)? (3) How do platforms differ in EEG content breadth and technical functionality (pediatric/neonatal content, intensive care unit [ICU]/intraoperative monitoring [IOM]/quantitative EEG [qEEG] coverage, and EEG scrolling/manipulation)?

### Identification of Platforms and Information Sources

From June 2025 to January 2026, we identified candidate platforms using a multipronged approach: (1) independent public web searches performed by multiple reviewers using common terms related to EEG education; (2) targeted screening of professional society and organizational websites relevant to EEG education and epilepsy/clinical neurophysiology training; (3) review of EEG education literature and conference-related materials; (4) platforms identified through author knowledge and professional networks; and (5) AI-assisted searching using ChatGPT (OpenAI) to generate additional candidate platform names and alternative search terms. The most recent search was completed in January 2026. Candidate platforms were compiled and deduplicated before eligibility assessment.

### Search Strategy

Web searches used English-language terms such as “EEG learning,” “EEG course,” “online EEG modules,” “EEG atlas,” “critical care EEG course,” and “epilepsy EEG curriculum.” Targeted website screening and review of literature and conference materials were conducted in parallel to capture resources not consistently indexed by general search queries. AI-assisted searching was conducted using ChatGPT (OpenAI) through iterative conversational queries to suggest additional EEG education-related search phrases and candidate online platforms. All suggestions underwent independent author review and eligibility assessment. Search strategy details are further outlined in eMethods.

### Eligibility Criteria

We defined an online EEG learning platform as a stable, web-based educational environment primarily intended to teach EEG interpretation or EEG-related clinical neurophysiology, with structured organization of content and at least 1 active learning feature beyond passive content consumption, such as knowledge checks, EEG case interpretation activities, interactive review tools, discussion-based learning, or live instructional engagement.

Platforms were eligible if they (1) were primarily designed to teach EEG interpretation or EEG-related clinical neurophysiology; (2) were available online; and (3) contained educational content suitable for clinicians in training, including neurology residents, epilepsy or clinical neurophysiology fellows, or equivalent trainees. Platforms were included regardless of cost, if core features could be ascertained from publicly accessible information.

Platforms were excluded if they consisted primarily of passive video lectures without EEG-focused learning components relevant to interpretation skills, lacked accessible EEG examples, or were not reasonably accessible for review (e.g., institution-only intranet resources). Standalone social media or video-sharing channels were excluded unless they functioned as part of a broader structured educational system. Because online educational content evolves rapidly, findings represent a snapshot of features available as of January 2026.

### Selection of Sources of Evidence

Each candidate platform underwent eligibility assessment based on publicly accessible webpages and available preview materials. The platform identification and selection process is summarized in Figure 1. Sixteen candidate online EEG educational sources were identified and assessed for eligibility. One standalone video-sharing channel was excluded because it did not meet the platform definition, leaving 15 eligible platforms for analysis.

**Figure:**
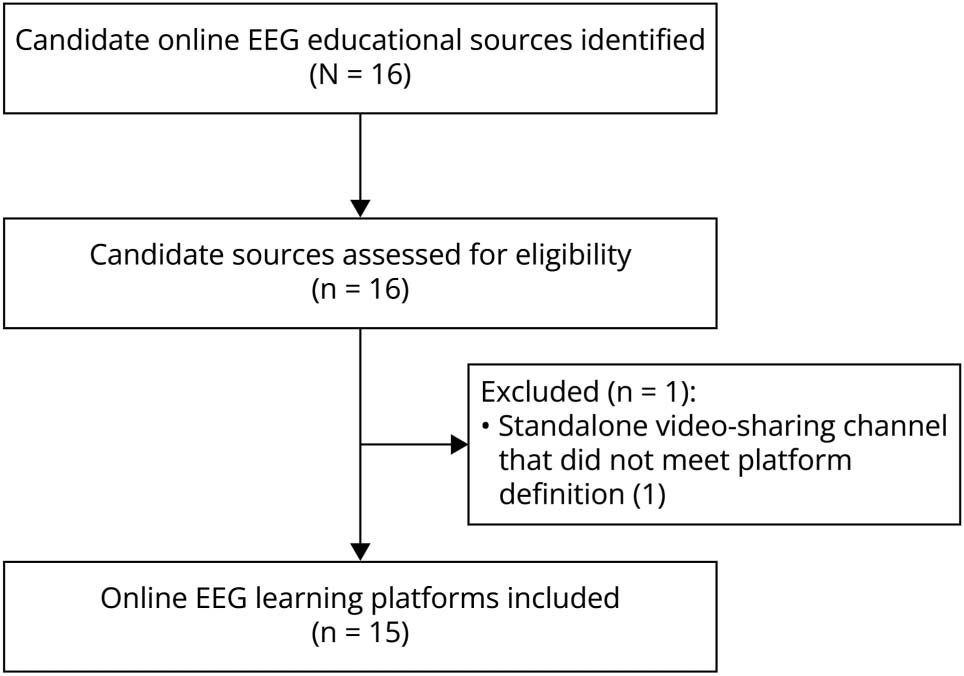
Identification and Selection of Online EEG Learning Platforms

### Platform Review and Data Charting Process

Each included platform was reviewed independently by 2 reviewers (Q.B.N. and G.K.) using a standardized abstraction form (eAppendix 1). The abstraction form captured platform access, curriculum structure, EEG content and technical features, assessment and feedback, and program-level integration. Reviewers examined publicly accessible content and documentation (e.g., landing pages, module outlines, sample lessons, publicly viewable cases, and preview materials). Discrepancies were resolved through discussion and consensus. For platforms requiring login or payment, reviewers extracted only information available without subscription, including features visible on public webpages, preview materials, or public-facing documentation. Features behind a paywall were not assessed.

### Data Items

We charted platform characteristics across 2 domains: (1) educational delivery and learning design and (2) EEG content breadth and technical interactivity.

Educational delivery and learning design included platform accessibility (free vs paid), account requirements, and the availability of learning formats such as long-form text resources, video lectures, static EEG images, and clinical vignettes. We also evaluated interactive learning features (e.g., discussion forums, live sessions, quizzes, and case discussions), assessment formats (multiple-choice questions [MCQs] and/or case-based interpretation exercises), feedback mechanisms (automated vs personalized or instructor feedback), and program integration, such as reporting learner progress to residency program directors.

### Creator Verification

To improve accuracy of feature characterization, we contacted free platform creators/administrators when contact information was available and invited them to complete a brief verification survey to confirm platform features and clarify access restrictions. Survey responses were used to verify or refine abstractions when discrepancies were identified.

### Critical Appraisal and Synthesis of Results

We did not conduct a formal critical appraisal of individual platforms because this scoping review aimed to map and describe available platform features rather than evaluate platform quality, educational effectiveness, or risk of bias. We summarized platform characteristics using descriptive tables and narrative synthesis, comparing platforms across educational delivery (interactivity, assessment, feedback, and program integration) and EEG technical functionality (content diversity, pediatric/neonatal representation, EEG review, and coverage of ICU/IOM/qEEG).

### Standard Protocol Approvals, Registrations, and Patient Consents

This study reviewed publicly available information about educational platforms and did not involve human participants, patient-level data, or identifiable private information. Institutional review board approval was not required.

### Data Availability

The search prompts with identification strategy, platform abstraction framework, survey questionnaire, and a sample of hybrid EEG curriculum are provided with the manuscript as eMethods, eAppendix 1, eAppendix 2, and eAppendix 3, respectively.

## Results

Sixteen candidate online EEG educational sources were identified and screened; 1 standalone video-sharing channel was excluded because it did not meet the platform definition, leaving 15 eligible platforms for analysis. No eligible platform was identified solely through artificial intelligence (AI)–assisted searching; all AI-generated candidates were corroborated through other sources before inclusion.

Table 1 summarizes educational delivery characteristics, including accessibility, format, interactivity, assessment, feedback, and program integration. Table 2 summarizes EEG content breadth and technical functionality among free platforms. Table 3 provides links to all platforms identified. Verification survey responses were received from representatives of 5 platforms: EEG Master, EEG Talk, EEG Platform, LearningEEG.com, and Yale EEG Modules, additional data are listed in eTable 1. Creator verification resulted in minor feature clarifications but did not change the overall conclusions of the review.

**Table 1:** Structured Overview of Online EEG Learning Platforms: Accessibility, Format, Interaction, Assessment, and Feedback

Platforms	Account needed	Long-form texts	Video lectures	Static images	Vignettes	Interaction	Type of assessment	Feedback provided	PD integration
Free									
AES EEG essentials and fellows curriculum	**+**		**+**		**+**		MCQ, case practice	Automated	
CCEMRC				**+**			MCQ	Automated	
EEG atlas online	**+**	**+**	**+**	**+**			Case practice	Automated	
EEG master	**+**	**+**		**+**			MCQ	Automated	
EEG pedia		**+**		**+**					
EEG talk			**+**	**+**		**+**			
EEG platform	**+**		**+**	**+**			MCQ	Automated	
ILAE roadmap to EEGs			**+**						
LearningEEG.com		**+**		**+**			MCQ	Automated	
Yale EEG modules			**+**	**+**			MCQ	Automated	
Paid									
AAN	**+**		**+**		**+**		MCQ	Automated	
ACNS bootcamp			**+**		**+**	**+**	MCQ	Personalized	**+**
ICNTN, epilepsy course	**+**		**+**			**+**	MCQ	Automated	
ILAE VIREPA EEG course	**+**		**+**		**+**	**+**	MCQ, case practice	Personalized	
USF/EEGCare course			**+**		**+**	**+**	MCQ, case practice	Personalized	

Abbreviations: AAN = American Academy of Neurology; ACNS = American Clinical Neurophysiology Society; AES = American Epilepsy Society; CCEMRC = Critical Care EEG Monitoring Research Consortium; ICNTN = International Child Neurology Teaching Network; ILAE = International League Against Epilepsy; MCQs = multiple-choice questions; USF = University of South FL; VIREPA = Virtual Epilepsy Academy.

Automated = system-generated feedback; Case practice = case-based interpretation exercises; Interaction = interactive features such as lecturer engagement, discussion forums, quizzes, case discussions, or live sessions; PD integration = course progress reported to a residency program director; Personalized = instructor-provided or expert-provided feedback tailored to learner responses.

+ = feature present. Blank cells indicate features not identified during review.

**Table 2:** EEG Content Breadth, Technical Functionality, and Curriculum Organization of Free Online Learning Platforms

Platforms	>50 EEGs	Pediatric/neonatal^a^	Video clips	EEG review^b^	ICU	IOM	QEEG	Curriculum organization^c^
AES EEG essentials and fellows curriculum		**++**	**+**	**+**	**+**	**+**	**+**	M
CCEMRC					**+**			T
EEG atlas online	**+**		**+**	**++**				T
EEG master	**+**	**++**		**++**	**+**		**+**	T
EEG pedia		**+**			**+**			T
EEG talk		**++**	**+**	**++**	**+**		**+**	M
EEG platform		**+**	**+**	**++**				M
ILAE roadmap to EEGs			**+**					T
LearningEEG.com		**++**						M
Yale EEG modules					**+**			T

Abbreviations: AES = American Epilepsy Society; CCEMR = Critical Care EEG Monitoring Research; ICU = intensive care unit; IOM = intraoperative monitoring; qEEG = quantitative EEG.

a Pediatric/neonatal: + = pediatric; ++ = pediatric and neonatal.

b EEG review: + scrollable/paginated EEG; ++ = scrollable/paginated EEG with manipulation functions, such as montage, filter, or amplitude adjustments.

c Curriculum organization: M = progressive modules; T = topic-based organization.

Blank cells indicate features not identified during review.

**Table 3:** Online EEG Learning Platforms Identified With Web Links

Platform	Link
AAN	aan.com/education/online-learning-programs
ACNS bootcamp	acns.org/education/cnp-bootcamp
AES EEG essentials and fellows curriculum	learn.aesnet.org/Listing/EEG-Essentials-Fundamental-Curriculum-10793
CCEMRC	acns.org/research/critical-care-eeg-monitoring-research-consortium-ccemrc
EEG atlas online	eegatlas-online.com/index.php/en/
EEG master	eegmaster.com
EEG pedia	eegpedia.org/index.php?title=Main_Page
EEG talk	eegtalk.com youtube.com/@F%C3%A1bioA.Nascimento
EEG platform	eegplatform.com
EEGucation YouTube channel (excluded)	youtube.com/user/EEGucation
ICNTN, epilepsy course	icnapedia.org/icntn-modules/modules/epilepsy
ILAE roadmap to EEGs	ilae.org/news-and-media/news-about-ilae/epileptic-disorders-roadmap-to-eegs
ILAE VIREPA EEG course	ilae.org/education/virtual-epilepsy-academy-virepa
Learning EEG	learningeeg.com
USF/EEG care course	eegcare.com/fellowship.htm
Yale EEG modules	campuspress.yale.edu/eegmodules/ youtube.com/@jmoeller78

Abbreviations: AAN = American Academy of Neurology; ACNS = American Clinical Neurophysiology Society; AES = American Epilepsy Society; CCEMRC = Critical Care EEG Monitoring Research Consortium; ICNTN = International Child Neurology Teaching Network; ILAE = International League Against Epilepsy; USF = University of South FL; VIREPA = Virtual Epilepsy Academy.

Across platforms, free resources were generally easy to access and emphasized self-directed learning through text, videos, static EEG examples, or case-based materials. More individualized features, including expert feedback and learner progress tracking, were uncommon and were mainly identified among paid platforms. Society-affiliated and academic platforms tended to provide structured foundational content, whereas platforms with greater interactivity or personalized feedback were less often freely available.

### Educational Delivery and Learning Design

Of the 15 platforms, 10 (67%) were free to access, whereas 5 (33%) required payment. Feedback was available on 12 platforms (80%): automated feedback on 9 platforms (60%) and personalized expert feedback on 3 platforms (20%). Notably, all platforms offering personalized feedback required payment. Only 1 platform (7%) reported trainee progress to a residency or fellowship program director and required a paid subscription.

These findings suggest that most available platforms can support broad exposure to foundational EEG concepts, but fewer replicate higher-level training functions such as supervised interpretation, individualized feedback, technical EEG manipulation, or program-level competency tracking.

### EEG Content Breadth and Technical Functionality

Among the 10 free platforms, all included adult EEG examples. Pediatric EEG content was available on 7 platforms (70%) and neonatal EEG content on 4 (40%). EEG manipulation was available on 4 platforms (40%). ICU/critical care EEG content was available on 6 (60%), qEEG content on 3 (30%), and IOM content on 1 (10%).

In summary, across free platforms, adult routine EEG content was consistently represented, while pediatric, neonatal, ICU, qEEG, and IOM content were less uniformly available. Technical interactivity was also limited, with fewer than half of free platforms allowing EEG manipulation.

## Discussion

In this scoping review, we evaluated publicly accessible online EEG learning platforms relevant to neurology resident and fellow education. These platforms collectively offer an accessible foundation for EEG interpretation, but they vary substantially in content breadth, interactivity, and feedback mechanisms.

The main strengths of current online EEG platforms are accessibility, flexibility, and scalability. Most platforms are asynchronous, allowing trainees to learn at their own pace and revisit material over time, and many are freely accessible. These features make them particularly attractive as supplemental tools for programs with limited local EEG volume, limited faculty teaching time, or variable curricular structure. This potential role is consistent with previous literature suggesting that e-learning, real-time online teaching, and virtual learning can improve EEG-related performance and may be especially useful in settings where educational resources are constrained.^9^

Many platforms also provide structured introductory materials, including long-form text, video lectures, static EEG examples, and case-based exercises, which may help standardize exposure to foundational concepts. An additional advantage of online resources is that they can aggregate examples of uncommon but educationally important EEG findings that trainees may not reliably encounter during a limited rotation. This may help address one of the central problems described in prior residency surveys: local variability in EEG exposure.^1,2^ In this way, online platforms may complement competency-based efforts that seek to ensure exposure to “must-know” EEG findings during residency.^3^

Despite these strengths, several limitations likely reduce the educational effect of currently available platforms. First, content breadth remains uneven. Adult routine EEG content predominates, whereas pediatric and neonatal EEG are less commonly represented. Similarly, ICU EEG, qEEG, and IOM are available on only a minority of platforms despite their growing relevance in modern neurologic practice. This mirrors previous literature showing that residency EEG education itself is variable and that important domains, including ICU EEG, may be insufficiently addressed.^1,9^

Second, clear curriculum structure and milestone alignment were uncommon among free platforms. Among free resources, American Epilepsy Society was the only platform we identified with a clear level-based order. Most other free platforms provided useful topic-based content but did not seem to organize learning by trainee level, competency milestone, or progression from basic to advanced EEG interpretation. Because we did not have full access to all paid platforms, some structured features behind paywalls may have been missed. This gap may make it harder to use online EEG platforms in competency-based residency or fellowship curricula and highlights the need for platforms that link EEG content to trainee progress and objective assessment.

Third, technical interactivity remains limited. Most resources rely on static screenshots or brief clips rather than full EEG viewers that allow learners to scroll through studies, adjust montages, manipulate filters, and replicate the workflow of real clinical interpretation. Because EEG interpretation is not only a pattern-recognition task but also a dynamic process of review, comparison, and artifact assessment, these limitations may hinder the translatability of online learning to clinical performance.

Fourth, feedback and assessment remain underdeveloped. Although many platforms include quizzes or case-based questions, these assessments are usually limited to multiple-choice or short-answer formats and often provide only automated feedback. Personalized expert feedback, especially on full tracing interpretation or report generation, is uncommon and seems more often in paid resources. This is important because previous work in residency EEG education has consistently identified the lack of objective and standardized measures of competency as a major gap.^1,2^ Moreover, current competency-based literature suggests that broad milestones alone are insufficient because they do not specify which EEG findings trainees should master.^3,13,14^ Although online quizzes may support knowledge acquisition, it remains unclear whether multiple-choice assessments, even when mapped to competencies, can adequately evaluate real-world EEG interpretation skills such as artifact discrimination, synthesis across an entire recording, and construction of an accurate EEG report. Recent review data similarly found that assessment methods across EEG education studies are heterogeneous, often not designed specifically for program evaluation, and lack standardized postcurriculum measures and long-term follow-up.^9^

Finally, program-level integration is minimal. Few platforms are designed to share learner progress with training program leadership or to align directly with milestone-based curricula. As a result, online resources may be difficult to incorporate into structured competency-based training pathways.

For training programs, the most impactful limitations are those that affect how learners practice and refine EEG interpretation. Features that allow learners to adjust EEG displays and receive individualized guidance should be prioritized because they support active skill development and error correction. Content expansion in more specialized areas remains valuable but may be a secondary priority for foundational resident and fellow education.

Traditional EEG education is still largely apprenticeship-based, with trainees interpreting studies under expert supervision, receiving real-time feedback, and gradually developing autonomy. Previous literature suggests that this supervised model remains important, particularly because time-limited rotations and lectures alone may be insufficient to develop proficiency.^9^

Our findings suggest that online platforms are best viewed as complements to this model rather than replacements for it. They may be especially helpful for standardizing foundational teaching, enabling deliberate practice outside the reading room, and broadening exposure to less common findings that a trainee may not otherwise encounter during a routine EEG rotation. Educational initiatives may improve EEG performance, but no single course can replace hands-on EEG reading, and the minimum requirements for competency remain incompletely defined.^15^ Accordingly, online platforms are unlikely to fully substitute for supervised clinical interpretation, particularly for nuanced pattern recognition, artifact identification, workflow-based review, and report generation. Deliberate practice requires feedback, correction, and refinement; without expert review, unsupervised practice may reinforce inaccurate interpretation habits.^12^

For residency and fellowship programs, these findings support a hybrid approach. Online platforms can serve as preparatory and longitudinal learning tools within a flipped-classroom model, providing core didactic content and opportunities for self-directed case exposure before or alongside faculty-supervised EEG review. This approach is consistent with previous EEG education work using short video-based EEG modules for residents to review before applying these concepts during EEG reading sessions.^16^ Such a model may be especially valuable for programs with limited EEG volume, limited subspecialty faculty time, or inconsistent access to pediatric, neonatal, or ICU EEG. A customized EEG curriculum that integrates online platforms and is tailored to local resources may improve efficiency for learners while helping address gaps in training. This use of e-learning as a supplement is also consistent with proposed solutions from prior program director surveys.^1,2^

However, programs should be cautious not to equate platform completion or quiz performance with EEG competency. Existing literature indicates that EEG competence requires clearer standards, better-defined learning expectations, and more objective evaluation methods than are currently used in many programs.^1,3^ Online resources may help fill exposure and content gaps, but they do not by themselves solve the broader problem of competency-based assessment. Programs may therefore need to pair online learning with supervised interpretation, milestone-informed case review, and structured local assessment of full-study interpretation and reporting.

These implications may extend beyond the United States. Because web-based resources can be distributed across institutions and countries, online platforms may also help support training in settings where access to local EEG educators or subspecialty expertise is limited. Previous work has similarly suggested that resource sharing through e-learning modules and online simulators may be valuable in resource-limited settings and may support broader international standardization efforts.^9^

Future directions in online EEG education can be considered at the level of institutions, learners, and platform development. For institutions, the next step is not simply to identify available resources, but to deliberately review and integrate them into local curricula and evaluation systems. Programs may benefit from selecting platforms that complement local strengths and address local gaps, then embedding them into structured teaching, supervised EEG review, and competency-based assessment. In this way, online resources can serve as part of a hybrid curriculum rather than as isolated supplemental tools. A sample of a hybrid EEG curriculum is provided in eAppendix 3.

For learners, online EEG platforms may be most useful when used strategically to address gaps in local training. Trainees can use these resources for self-directed learning in areas with limited exposure, such as pediatric, neonatal, ICU, or less common EEG patterns, and to reinforce concepts encountered during supervised interpretation. This may be especially valuable in programs where EEG volume, faculty time, or case diversity is limited.

EEG education shares features with other visually intensive disciplines such as electrocardiogram (EKG) interpretation, radiology, and pathology, where skill develops through repeated exposure to representative patterns, annotated examples, and feedback. However, unlike many static image-based disciplines, EEG interpretation requires dynamic review of a time-based signal, including scrolling, montage adjustment, filter manipulation, artifact recognition, and clinical synthesis. Ideally, future platforms would function as cloud-based repositories in which users could upload deidentified EEG studies, scroll through recordings from beginning to end, manipulate montages and filters, and interpret tracings under conditions that better reflect clinical uncertainty. This would more closely approximate authentic EEG reading than posttest surveys or MCQs, which cannot fully capture the complexity of interpreting a full-length study in real time. Advanced AI may have a dual role in the next generation of EEG education platforms. First, AI could support platform development by organizing large repositories of deidentified EEGs, generating preliminary structured interpretations, identifying patterns in learner error, and recommending targeted educational content. In an ideal model, learners could review cloud-based EEG cases that mimic real clinical interpretation, submit their reports, and receive real-time feedback generated through an AI comparison with expert-reviewed interpretations. Second, AI may support adaptive EEG education by monitoring learner performance longitudinally, identifying areas of persistent difficulty, and directing trainees toward individualized practice resources, cases, or instructional modules aligned with their evolving needs. However, these applications require careful oversight, including robust deidentification, expert validation, peer review of AI-generated reports, data governance, and sustainable infrastructure.

Over time, platforms with these capabilities could support more standardized assessment across institutions by benchmarking interpretation skills using shared cases and structured scoring methods. Such systems might help programs determine whether trainees have achieved expected interpretive competence and could also support periodic reassessment after graduation to maintain EEG interpretation skills—an area of growing need.^17^ They could also allow programs to compare their learners, at all stages of training, with learners at the same levels at other institutions, helping identify relative strengths and weaknesses in local curricula.

A formal protocol was developed by the study team but was not prospectively registered. This represents a methodologic limitation because the absence of protocol registration limits external verification that the review scope, eligibility criteria, and charting domains were not modified after platform identification. Our review was limited to publicly accessible English-language information and creator-provided clarifications; paid content was not comprehensively reviewed. As a result, some EEG content breadth and technical functionality metrics, including subspecialty content, case volume, EEG scrolling, montage/filter adjustment, and other interactive features, may have been underestimated for platforms with content behind login requirements or paywalls. Restricting the review to English-language platforms may also have excluded relevant EEG education resources developed for non–English-speaking learners. In addition, creator verification responses were available for only a subset of platforms, raising the possibility of response bias if responding platforms differed systematically from nonresponding platforms. We did not calculate formal interrater reliability metrics for platform abstraction. Although 2 reviewers independently reviewed platform features and resolved discrepancies by consensus, the absence of kappa statistics or percent agreement limits our ability to quantify reproducibility of the charting process. Some platforms are extensive, and complete review of all content may not have been feasible, raising the possibility of misclassification of less visible features. Platform features evolve rapidly, and our findings represent a snapshot as of January 2026. Finally, because our goal was to map platform characteristics, we did not evaluate educational effectiveness or learner outcomes.

In conclusion, publicly accessible online EEG learning platforms can broaden access to foundational EEG education, but they vary widely in interactivity, feedback, and content diversity. Key gaps include limited pediatric/neonatal content; learner inability to manipulate EEG parameters such as montage, filters, amplitude, and display settings; and sparse personalized feedback or program-level integration. Hybrid models that combine online learning with supervised interpretation and feedback are likely to maximize educational effect for resident and fellow training.

## Glossary

AI: artificial intelligence
ICU: intensive care unit
IOM: intraoperative monitoring
MCQ: multiple-choice question
qEEG: quantitative EEG
